# Linkage maps of the Atlantic salmon (*Salmo salar*) genome derived from RAD sequencing

**DOI:** 10.1186/1471-2164-15-166

**Published:** 2014-02-27

**Authors:** Serap Gonen, Natalie R Lowe, Timothé Cezard, Karim Gharbi, Stephen C Bishop, Ross D Houston

**Affiliations:** 1 The Roslin Institute, University of Edinburgh, Midlothian EH25 9RG, Scotland, UK; 2 Edinburgh Genomics, Ashworth Laboratories, King’s Buildings, University of Edinburgh, Edinburgh EH9 3JT, Scotland, UK

## Abstract

**Background:**

Genetic linkage maps are useful tools for mapping quantitative trait loci (QTL) influencing variation in traits of interest in a population. Genotyping-by-sequencing approaches such as Restriction-site Associated DNA sequencing (RAD-Seq) now enable the rapid discovery and genotyping of genome-wide SNP markers suitable for the development of dense SNP linkage maps, including in non-model organisms such as Atlantic salmon (*Salmo salar*). This paper describes the development and characterisation of a high density SNP linkage map based on SbfI RAD-Seq SNP markers from two Atlantic salmon reference families.

**Results:**

Approximately 6,000 SNPs were assigned to 29 linkage groups, utilising markers from known genomic locations as anchors. Linkage maps were then constructed for the four mapping parents separately. Overall map lengths were comparable between male and female parents, but the distribution of the SNPs showed sex-specific patterns with a greater degree of clustering of sire-segregating SNPs to single chromosome regions. The maps were integrated with the Atlantic salmon draft reference genome contigs, allowing the unique assignment of ~4,000 contigs to a linkage group. 112 genome contigs mapped to two or more linkage groups, highlighting regions of putative homeology within the salmon genome. A comparative genomics analysis with the stickleback reference genome identified putative genes closely linked to approximately half of the ordered SNPs and demonstrated blocks of orthology between the Atlantic salmon and stickleback genomes. A subset of 47 RAD-Seq SNPs were successfully validated using a high-throughput genotyping assay, with a correspondence of 97% between the two assays.

**Conclusions:**

This Atlantic salmon RAD-Seq linkage map is a resource for salmonid genomics research as genotyping-by-sequencing becomes increasingly common. This is aided by the integration of the SbfI RAD-Seq SNPs with existing reference maps and the draft reference genome, as well as the identification of putative genes proximal to the SNPs. Differences in the distribution of recombination events between the sexes is evident, and regions of homeology have been identified which are reflective of the recent salmonid whole genome duplication.

## Background

The dramatic increase in the production of farmed fish in the past few decades has resulted in aquaculture species becoming of huge economic importance, promising a sustainable resource of high quality protein and long-chain fatty acids. Salmonids, in particular Atlantic salmon, are amongst the most important aquaculture species. In 2010, approximately 1.5 million tonnes of Atlantic salmon were produced from farms worldwide, corresponding to a value of just over $7.8 billion [1].

Salmonidae originate from a common ancestor whose genome underwent a duplication event around 25 - 100 million years ago (MYA) [2], with recent estimates from sequence data (nuclear and mitochondrial) suggesting a date of ~60 MYA [3]. Extant salmonid species have not yet fully recovered the diploid state, with areas of the genome still showing evidence of tetraploid segregation [4]. In female Atlantic salmon, all loci appear to segregate in a diploid fashion [5]. Although much of the male genome segregates disomically, secondary quadrivalent formation during meiosis between parts of chromosomes which are ancestrally homeologous has been described [5]. Homeologous pairing is thought to occur only at the telomeres since it takes place after homologous chromosome pairing, and has been postulated to be responsible for the distinct lack of recombination observed in male salmon [2,6]. While in most species the heterogametic sex often shows reduced recombination rates compared to the homogametic sex [7], the ratio in Atlantic salmon is one of the highest observed in vertebrates (up to 17:1) [8]. Existing linkage maps for Atlantic salmon also highlight a marked difference between the sexes in the distribution of putative crossover events; equal dispersion is observed along chromosomes in females, in contrast to telomere-specific recombination in males with little or no recombination at centromeric regions [9-11]. As a result, marker order and positions are more reliably estimated in female-specific recombination maps.

The genomic resources for Atlantic salmon are amongst the most extensive of all aquaculture species [12,13], and include several genetic maps, a physical map, an extensive EST database of approximately 500,000 tags and several microarrays [10,14-21]. The Atlantic salmon genome is also in the process of being sequenced and assembled [16], and the first draft assembly is available (NCBI Assembly GCA_000233375.1; http://www.ncbi.nlm.nih.gov/Traces/wgs/?val=AGKD01). However, the recent genome duplication and frequent long repeat regions are hampering the genome assembly [16]. The linkage maps currently available for Atlantic salmon include those based on amplified fragment length polymorphisms (AFLPs), microsatellites and more recently single nucleotide polymorphisms (SNPs) [9-11,22]. Microsatellites are generally most informative for linkage mapping due to their variability and multi-allelic nature. However, bi-allelic SNPs are increasingly utilised due to their abundance and amenity to accurate and high-throughput scoring (e.g. [23-25]). The most recent salmon linkage map by Lien at al. [11] is comprised of 5,650 SNP markers in 29 linkage groups. In contrast to other studies (for example [9,10,22]), the difference in overall map length between males and females was minor (1.38:1, male:female). A possible reason for this discrepancy is that the increased marker density resulted in the genotyping of more markers in the telomeric regions. Therefore, Lien et al. [11] suggested that overall average male and female recombination rates are similar, but the distribution of recombination events is markedly different, with male recombination events being more frequent at the telomeres, as supported by other Atlantic salmon linkage maps [5,9-11].

While dense SNP genotyping platforms are still in development for salmonid species, next generation sequencing (NGS) technologies are making the SNP discovery and genotyping process much more feasible, efficient, and cost-effective [26,27]. Several methods of genotyping-by-sequencing can be used to simultaneously discover and score thousands of SNP markers in pooled, barcoded samples of any species of interest [28]. For example, Restriction-site Associated DNA sequencing (RAD-Seq), first described by Baird et al. [29], has been applied to many organisms without a reference genome [30-33]. It has rapidly become a popular method in aquatic species for quick SNP discovery and genotyping for building linkage maps [34], population genomics [35,36], comparative genomics [37] and QTL mapping [38,39]. In addition to the SNP genotypes, paired-end RAD-Seq provides several hundred bases of flanking sequence from the assembly of paired-end reads of randomly sheared genomic fragments anchored at the same restriction site (paired-end contigs) [30]. These sequence data assist in whole genome sequencing of an organism via the assembly and ordering of genome contigs and scaffolds (for example [40]). RAD-Seq has also been applied to fine map QTL in Atlantic salmon [38], but a linkage map based on the SNP genotypes in the restriction enzyme site flanking sequences has not yet been constructed, and the location of the RAD-Seq-derived SNPs with respect to the Atlantic salmon reference genome contigs and putative genes is largely unknown.

The main aim of this study was to construct a high density SNP linkage map of the Atlantic salmon genome using SNP markers derived from a RAD-Seq analysis using the SbfI restriction enzyme. Additional aims, building on this linkage map, were to (i) investigate the differences in recombination rate and distribution between male and female maps; (ii) integrate the new RAD-Seq linkage map with existing linkage/physical maps and the draft Atlantic salmon reference genome; (iii) identify putative genes proximal to the SNPs in the linkage map using comparative genomics; and (iv) investigate the salmonid genome duplication by comparisons to rainbow trout and stickleback linkage groups.

## Results

### RAD sequencing

The Atlantic salmon samples used in this study were from the two SalMap reference families, for which dense sex-specific microsatellite linkage maps exist (ASalBase, [8,41]). Four unrelated individuals (two males and two females) sourced from the river Tay in Scotland, UK, were used as parents in the production of two full-sibling families (denoted Br5 and Br6). Each full-sibling family consisted of 46 F1 offspring, resulting in 96 individuals in the study (before filtering individuals for poor quality genotyping results). To maximise the chances of detecting segregating SNPs in the parents, the parents were sequenced at a substantially higher depth than the offspring (Additional file 1: Table SA1). The average sequence depth was 11 million reads per parent and 2 million reads per offspring, which was reduced to 3.5 million and 0.8 million following the removal of putative PCR duplicates (see Davey et al. [28]; Additional file 1: Table SA1). Paired-end RAD-Seq results in sequence from both ends of the randomly-sheared genomic fragments which are anchored at the SbfI cleavage sites. The resulting dataset includes a stack of ‘read 1’ corresponding to the 95 bp (following the removal of the nucleotide barcode sequence) immediately adjacent to the SbfI cleavage site (referred to as ‘RAD loci’ hereafter) and an associated paired-end contig resulting from the assembly of the ‘read 2’ (as described in Baxter et al. [30]; referred to as ‘PE contigs’ hereafter).

Following the merging of reads into RAD loci across individuals, 75,688 distinct RAD loci were detected (see Materials and methods) which is indicative of 37,844 SbfI cleavage sites in the Atlantic salmon genome. This number is comparable to a previous study by our group [38] in families of farmed Atlantic salmon, and indeed > 97% of the RAD loci observed in the current study were also observed in [38]. This demonstrates that SbfI RAD-Seq is sampling the same sites in the Atlantic salmon genome across wild and farmed populations, with positive implications for its reproducibility as a genotyping technique.

### SNP discovery, filtering, genotyping and validation

**Figure 1:**
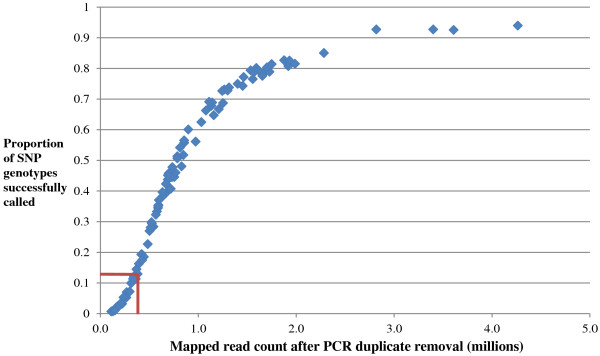
**Relationship between read depth and call rate.** The number of reads per individual following exclusion of PCR duplicates (x-axis) plotted against the proportion of SNP genotypes successfully called for all putative SNPs (y-axis). The red lines on the graph indicate the thresholds below which individuals were removed from the current analysis due to an excess of missing genotypes.

A total of 28,415 putative SNPs were discovered, of which 11,103 were detected in the RAD loci and 17,312 were detected in the PE contigs. Stringent filtering criteria (see Materials and methods and Table 1) were applied to identify and remove individuals and SNPs with a high level of missing data and/or Mendelian errors. 4,895 SNPs with a genotype pattern suggestive of paralogous sequence variants (PSVs) were removed (listed in Additional file 2). These are a useful resource for excluding PSVs in future SbfI RAD-Seq of Atlantic salmon and other salmonids. The proportion of missing genotypes of an individual was inversely related to the sequence coverage for that individual (Figure 1); this was due to the removal of genotypes at individual SNP loci where sequence depth was below the threshold chosen to ensure high confidence in the genotype call (see Materials and methods). As a result, 15 individuals were removed from further analysis (shown by red lines in Figure 1). It is clear that a read depth of *ca.* 1 million reads per individual (following removal of PCR duplicates) is required to ensure high levels of high confidence genotype coverage. A further 4 individuals were removed from further analysis due to a high Mendelian error rate (> 200 errors). Post-filtering, the number of SNPs retained for the construction of the linkage map was 8,257, and the total number of individuals remaining was 77 (2 parents and 36 offspring in family Br5; 2 parents and 37 offspring in family Br6).

**Table 1:** SNP and individual filtering procedure

**Filtering step**	**Number of SNPs eliminated**	**Number of SNPs remaining**	**Number of individuals eliminated**	**Number of individuals remaining**
Raw RAD-Seq processing	0	28,415	0	96
Missing Genotypes	14,778	13,637	0	96
SNPs (> 50%)				
Missing Genotypes	0	13,637	15	81
Individuals (> 25%)				
Excess heterozygosity	4,895	8,742	0	81
(PSVs; > 70%)				
Mendelian errors	485	8,257	0	81
SNPs (≥ 2)				
Mendelian errors	0	8,257	4	77
Individuals (> 200)				

Stringent quality control filtering was applied to the initial set of 28,415 putative SNPs generated from the raw RAD-Seq reads. Filtering parameters for SNPs included removing excess missing data (> 50%), excess Mendelian errors (≥ 2 individuals) and excess heterozygosity (putative PSVs; > 70%). The final number of SNPs left for linkage map construction was 8,257. Individuals were removed if they showed excess missing genotypes (> 25% of the SNPs) and/or excess Mendelian errors (> 200 SNPs). 77 individuals remained for linkage map construction post-filtering.

To validate a subset of the RAD-Seq-derived SNPs and to compare genotype calls with a more established genotyping technology, a subset of 47 SNPs were genotyped across the mapping panel using KASP technology (LGC Genomics, Herts, UK) (Additional file 3). On average, a 97% correspondence between KASP and RAD-Seq genotypes was found (Additional file 3). Given the complex, duplicated nature of the salmon genome, no genotyping assay is likely to have complete accuracy across all SNPs. Therefore, 97% concordance between genotype calls using two disparate technologies suggests that the RAD genotyping described herein is reliable.

### Linkage map construction

Following the SNP filtering process, the linkage arrangements between the remaining 8,257 putative SNPs were assessed. Anchor markers were used to assign SNPs to each of the 29 groups (details of anchor markers are given in Additional file 1: Table SA2). 6,458 SNPs were initially assigned to a linkage group using CRI-MAP software (version 2.4 [42], modified by Xuelu Liu (Monsanto)) (Table 2, column 3), with an average of 220 SNPs per linkage group. 5,787 of the SNPs were from the RAD loci and a further 671 were from the PE contigs. The lower number of PE SNPs is likely to be due to the lower sequence coverage of PE contigs compared to the RAD loci. In family Br5, 3,640 markers showed informative segregation patterns (i.e. were either: heterozygous in the mother and fixed in the father or heterozygous in the father and fixed in the mother). The corresponding number in family Br6 was 3,699 (Table 3).

**Table 2:** Number of SNPs assigned to linkage groups

**Atlantic salmon linkage group**	**Atlantic salmon chromosome**	**Number of SNPs on linkage group (CRI-MAP)**	**Final number of SNP markers ordered on each linkage group (Onemap)**			
			**Br5 mother**	**Br6 mother**	**Br5 father**	**Br6 father**
1	2	244	59	23	73	68
2	10	350	102	86	88	79
3	14	197	31	35	68	76
4	6	283	47	43	84	79
5	13	306	67	84	84	85
6	12	257	72	46	81	71
7	24	138	27	47	51	39
8	15	520	78	47	69	84
9	11	226	50	64	67	49
10	9	394	95	94	113	103
11	3	336	48	92	98	103
12	5	224	24	29	74	68
13	19	197	49	58	43	59
14	21	152	38	40	47	33
15	27	132	31	39	40	43
16	18	209	33	58	65	67
17	1	442	70	78	130	129
18	23	155	36	33	55	51
19	8	42	8	0	13	15
20	25	115	20	33	26	23
21	26	113	25	25	30	35
22	17	158	19	44	33	64
23	16	215	58	49	66	55
24	7	169	22	17	57	56
25	20	237	65	77	62	73
28	4	220	19	32	80	83
30	29	116	43	23	36	37
31	28	132	34	37	42	39
32	22	179	41	66	58	51
**TOTAL**	**-**	**6,458**	**1,311**	**1,402**	**1,833**	**1,817**

SNPs were assigned to a linkage group using previously mapped anchor markers in CRI-MAP. SNPs were then ordered on each linkage group in Onemap for each individual mapping parent separately.

**Table 3:** Number of SNPs showing sex-specific segregation patterns and the total map length for each mapping parent

**Mapping parent**	**No. of segregating SNPs**	**Total map length (cM)**
Br5 mother	1,688	2,807
Br5 father	1,952	2,170
Br6 mother	1,804	2,358
Br6 father	1,895	1,426

6,458 SNP markers were assigned to a linkage group using the CRI-MAP software. The table gives the total number of markers segregating for each parent from this subset of assigned markers.

**Figure 2:**
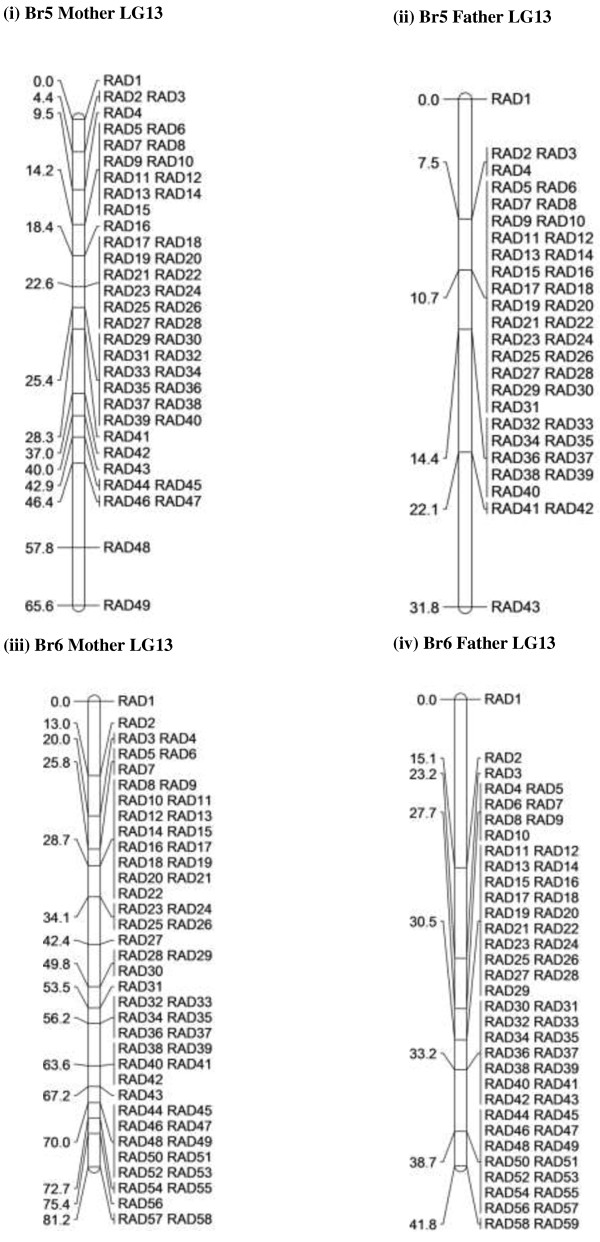
**Example linkage maps.** Maps for linkage group 13 for: **(i)** Br5 mother; **(ii)** Br5 father, **(iii)** Br6 mother; **(iv)** Br6 father. Map lengths are shorter in the male parent maps and markers are more widely spaced in the female parent maps. Marker names on the drawn maps are coded as RAD1-RADX depending on the ordered position of the marker on the linkage group.

Individual linkage maps were then constructed using the Onemap software [43] (modified by Marcelo Mollinari, Department of Genetics, University of São Paulo) for each parent using the sex-specific segregating markers only. The numbers of SNPs positioned and ordered on each linkage group are given in Table 2 (columns 4 - 7). Figure 2 shows linkage maps drawn for all four parents for an example linkage group (LG 13). All individual parent maps with ordered SNPs and positions are given in Additional file 4. No linkage map could be constructed for linkage group 19 for the female parent of family Br6 due to very few female-segregating markers being assigned to this linkage group.

### Distribution of recombination events across the genome

One of the striking features of the salmon genome is the large difference in recombination rate and distribution observed between the sexes [5,8-11]. To investigate this phenomenon using the RAD-Seq linkage map, the map lengths for each linkage group were compared for each parent within a family to give an indication of the male:female recombination ratio (Additional file 1: Table SA3). For family Br5 the overall map lengths between the sexes were similar, with a female map length of 2,807 centiMorgans (cM) and a male map length of 2,169 cM, giving a recombination ratio of 1:1.3. This similarity of map length was generally consistent across most linkage groups, although for linkage groups 2, 21 and 31 the female map was significantly longer (ratio > 1:3; Additional file 1: Table SA3). For family Br6 the female map length was 2,358 cM and the male map length was 1,426 cM giving a male:female ratio of 1:1.7. The larger ratio and smaller male map observed in family Br6 is likely related to two features of the male parent map. Firstly, the markers on linkage group 31 all clustered at 0 cM thus no ratio could be calculated. Secondly, linkage group 9 in family Br6 showed an extreme male:female map distance ratio of 1:10.

**Figure 3:**
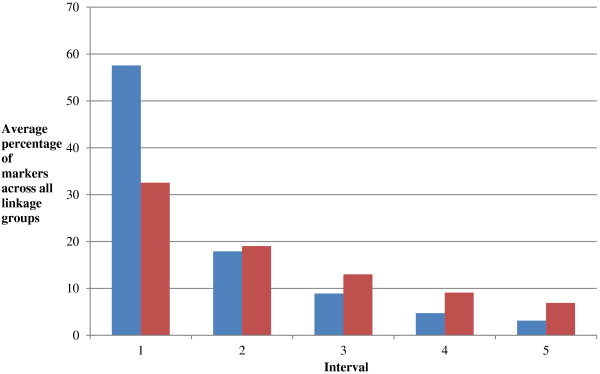
**Comparison of marker clustering between male and female linkage maps.** For each linkage group for each parent, the five intervals with the highest percentage of markers were identified. For each of these intervals, an average percentage of markers was calculated across all linkage groups and both families Br5 and Br6. Blue bars = Male parent average percentages; Red bars = Female parent average percentages. A greater clustering of markers in interval one is apparent in male parents.

In addition to the overall heterochiasmy in salmonids, previous studies have presented evidence for major differences in the distribution of putative recombination events between males and females, with male recombination events thought to cluster towards the telomeres [10,11]. To compare the distribution of putative recombination events in the current study, for each linkage group in each family the shortest parental map was identified and split into 5 cM intervals. The longer map (derived from the parent of the opposite sex) was then split into an equal number of evenly sized intervals. The five intervals containing the most SNPs were identified for each map, and an overall average of the percentage of markers in the top five most populated intervals was calculated and compared for the two sexes across both families Br5 and Br6 (Figure 3; see Materials and methods). It is evident that markers are much more clustered into one or two peaks of marker density in males, compared to females. This corresponds to a putative recombination desert, postulated to be at the centromeric regions of chromosomes in males. Conversely, fewer markers were found in intervals closer to the extremes (putative telomeres) of male linkage groups. For example for family Br5, the average percentage of markers located at the extremes of the linkage groups was 19% in females vs. 8% in males. This suggests more frequent recombination events in putative telomeric regions in males, in line with previous salmonid linkage mapping studies [9-11,22].

### Integration of the RAD-Seq maps with the Atlantic salmon reference genome

Flanking sequences for the mapped RAD-Seq SNPs (RAD loci and PE contigs) were aligned to the Atlantic salmon draft reference genome sequence contigs ([16]; NCBI Assembly GCA_000233375.1; http://www.ncbi.nlm.nih.gov/Traces/wgs/?val=AGKD01). This allowed the assignment of 4,367 Atlantic salmon reference genome contigs (corresponding to 57 Mb of sequence) to at least one linkage group (Table 4; Additional file 5). It is noteworthy that 110 genome sequence contigs showed significant sequence similarity to two different linkage groups, and two contigs to three linkage groups, which is indicative of homeology resulting from the recent genome duplication (Additional file 6; Table 5). For example, 25 contigs aligned to both Atlantic salmon linkage groups 4 and 11. Homeology between these two linkage groups has also been inferred in other studies [6,8,11,44].

**Table 4:** Reference genome contigs assigned to Atlantic salmon linkage groups

**Atlantic salmon linkage group**	**Atlantic salmon chromosome**	No. of genome contigs*	Amount of sequence data (kb)**	**Stickleback linkage group**	**Rainbow trout linkage group**
1	2	177	2,030	20	***2/27/*** *29/31*
2	10	262	3,410	19	***6/*** *8/27*
3	14	156	2,170	3/10	*3/****23/*** **29**
4	6	230	2,880	11	**2/ *9/ *** **24**
5	13	209	2,530	NA	*9/22*
6	12	211	3,090	17/9	*2/29*
7	24	94	1,160	13	*10*
8	15	175	2,220	18	*21/***23**
9	11	180	2,160	2	*1/****10/*** *18*
10	9	298	3,850	1	***3/*** **4/***25/26*
11	3	232	3,480	11/3	***2/*** *9/13*
12	5	173	2,110	20	*3/27/****31***
13	19	110	1,500	21/5	***17/*** *19/22*
14	21	111	1,570	16	***5/*** *31*
15	27	118	1,330	10/20	*16*
16	18	159	1,900	6	*6/***21**
17	1	254	3,780	14/6	*8/14/30*
18	23	103	1,300	8	*24*
19	8	33	454	NA	***14/*** *20*
20	25	60	809	16	*31*
21	26	97	1,330	2	*10/****18***
22	17	125	1,530	NA	*7/12/****15***
23	16	164	2,210	19	*6/16/****27***
24	7	109	1,370	4	***7/*** *15*
25	20	196	3,100	13	*11/19*
28	4	165	2,200	7	*14/20*
30	29	76	992	21	*7/****17***
31	28	84	1,130	5	*17/****22***
32	22	120	1,520	17	*12*
**Genome wide**	**-**	**4,367**	**57,402**	**-**	**-**

Atlantic salmon reference genome contigs were assigned to linkage groups by Blastn alignment against mapped RAD contigs. Column 5 shows the stickleback linkage groups orthologous to the Atlantic salmon linkage groups identified by this study. Column 6 shows the Atlantic salmon - rainbow trout orthologous linkage groups as defined by Phillips et al. [44] (bold) and Danzmann et al. [6] (bold italic) individually, and those identified in both studies (italic). 50% of the stickleback - rainbow trout relationships suggested in this table have been identified in another study comparing rainbow trout and stickleback only [45].

*Grand total includes genome contigs assigned to more than one linkage group only once, thus is less than the sum of genome contigs per linkage group.

**Grand total given includes sequence data of genome contigs assigned to more than one linkage group only once, thus is less than the sum of sequence (kb) assigned per linkage group.

**Table 5:** Homeology between linkage groups

**Atlantic salmon Linkage groups**	**Atlantic salmon Chromosomes**	**No. of shared contigs**	**Danzmann et al. [**[6]**] correspondence**
4/11§*	6/3	25	E
10/25	9/20	11	G/H
1/12§*	2/5	9	B
9/21*	11/26	9	J
3/11	14/3	8	M
22/24§*	17/7	8	K
1/6§*	2/12	5	D
3/15	14/27	4	B
2/23	10/16	3	M,J/K
2/18	10/23	3	M
7/25	24/20	3	I
19/28§*	8/4	3	-
5/17	13/1	2	I
9/28	11/4	2	G/H
3/14	14/21	1	-
5/28	13/4	1	G/H
6/32§	12/22	1	L
16/17§	18/1	1	D
17/31	1/28	1	D
22/23§*	17/16	1	K
22/30	17/29	1	K
17/32	1/22	1	-
12/17	5/18	1	-
10/32	9/22	1	-
9/25	11/20	1	G/H,I
4/6	6/12	1	-
2/25	10/20	1	-
2/6	10/12	1	-
9/14	11/21	1	-
3/13/17	14/19/1	1	3&13 = M
1/6/32	2/12/22	1	1&6 = D
**TOTAL**	**-**	**112**	**-**

112 Atlantic salmon reference genome contigs showed alignment to two or more linkage groups. The number of times two or more Atlantic salmon linkage groups shared an Atlantic salmon genome contig was counted, and is presented in column three. Column four shows the proto-Acinopterygian ancestral linkage group shared between Atlantic salmon linkage groups in column one, as defined by Danzmann et al. [6].

§ Also identified in [8]; *Also identified in [44].

### Comparative genomics: identification of RAD-marker-associated genes

A two-stage strategy was employed to identify genes associated with the mapped and ordered RAD-Seq SNPs. In stage one, the repeat-masked flanking sequence of the mapped RAD-Seq SNPs (including both the RAD locus and the PE contig; hereafter referred to as “mapped RAD contigs”) was screened for sequence similarity to all known three-spined stickleback (*Gasterosterus aculeatus*) gene sequences using tblastx. The stickleback was chosen because it is one of the most closely-related species to Atlantic salmon for which there is a near-complete annotated reference genome sequence. Significant sequence similarity (E-value < 1e^-5^) between mapped RAD contigs and stickleback genes was observed for approximately 17% of the mapped RAD contigs (Table 6).

**Table 6:** RAD-Seq SNPs located proximal to a putative gene

**Parent**	**No. of mapped SNPs**	**No. of gene-associated SNPs (Stage 1)**	**Percentage of gene-associated SNPs (Stage 1)**	**No. of gene-associated SNPs (Stage 2)**	**Percentage of gene-associated SNPs (Stage 2)**
Br5 Mother	1,311	212	16.2	541	41.3
Br6 Mother	1,399	227	16.2	621	44.4
Br5 Father	1,833	327	17.8	843	46.0
Br6 Father	1,817	298	16.4	815	44.9

The number of RAD-Seq SNPs located within or close to genes based on direct alignment of mapped RAD contigs (Stage 1; columns three and four) or Atlantic salmon reference genome contigs (Stage 2; columns five and six) against the stickleback nucleotide gene sequences downloaded from Ensembl BioMart (http://www.ensembl.org/biomart/martview/, Database = Ensembl Genes 72, Dataset = *Gasterosterus aculeatus* genes (BROADS1)). Column two gives the total number of sex specific segregating SNPs which were ordered and positioned within a linkage group using the Onemap software.

**Figure 4:**
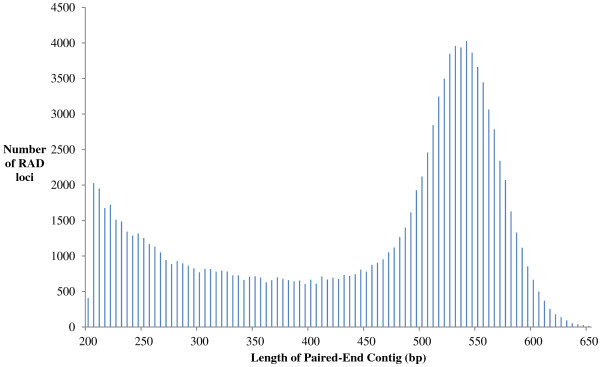
**Distribution of RAD paired-end contig lengths.** Paired-end (PE) RAD-sequencing generates a read from the P1 adaptor which is located at the restriction site and a PE contig generated from sequencing at the P2 adaptor at the sheared end of the fragment. The most frequent length of the PE contigs was between 450 and 600 bp. A small number of PE contigs were over 650 bp in length; these are not shown in the figure.

However, the mapped RAD contigs are relatively short (95 bp for RAD loci; 450 – 600 bp for PE contigs; Figure 4) and therefore genes close to, but not within, the mapped RAD contigs may be undetected. Therefore, in stage 2, the 4,367 Atlantic salmon reference genome contigs assigned to a linkage group (as described above) were repeat-masked and aligned (tblastx) with the stickleback gene sequences. Significant sequence similarity to stickleback genes was observed for 2,840 contigs (65%), of which 80 aligned to two Atlantic salmon linkage groups. In total, ~50% of the mapped SNPs were associated with a putative gene (Table 6 and Additional file 4). On average, 70% of the genes identified in stage 1 for each linkage group were also identified in stage 2. Overall, across all individuals and all linkage groups, Atlantic salmon orthologs for 2,030 stickleback genes were identified and mapped. These data may increase the utility of the RAD-Seq SNPs for QTL mapping and subsequent identification of candidate genes.

### Comparative genomics: synteny analysis

To investigate regions of conserved synteny (orthology) between the Atlantic salmon and stickleback genomes, the linkage group positions of the genes associated with the mapped RAD contigs in the stickleback reference genome were recorded. For each of the salmon linkage groups, the stickleback linkage groups to which the mapped RAD contigs most frequently aligned to was identified (see Materials and methods). Regions of conserved synteny were identified for 26 of the 29 Atlantic salmon linkage groups. However, no clear pattern of orthology with a stickleback linkage group was observed for Atlantic salmon linkage groups 5, 19 and 22 (chromosomes 13, 8 and 17 respectively) (Table 4, column 5).

### Comparative genomics: genome duplication

As described above, 110 and 2 Atlantic salmon genome contigs showed significant alignment to two and three Atlantic salmon linkage groups respectively (Table 5). In total, 31 homeologies were identified. 22 of these have been shown to originate from the same proto-Acinopterygian ancestral karyotype as defined by Danzmann et al. [6]. In order to confirm these homeologies, the Atlantic salmon linkage groups which share a single stickleback linkage group were identified from Table 4, and this is summarised in Additional file 1: Table SA4. Based on this, 12 homeologies were identified, 10 of which had already been identified in Table 5 using shared genome contig assignments. To test the theory of a salmonid specific duplication, orthologous relationships between Atlantic salmon and rainbow trout relative to stickleback linkage groups were analysed using previously published data [6,8,44]. A 1:1 correspondence between single Atlantic salmon linkage groups and rainbow trout linkage groups was not observed (Table 4). For 10 of the 12 Atlantic salmon homeologies identified due to sharing a single stickleback linkage group (Additional file 1: Table SA4), two rainbow trout linkage groups were identified.

## Discussion

### Linkage map construction

We have constructed and characterised a high density RAD-Seq-derived SNP linkage map in Atlantic salmon. As RAD-Seq becomes increasingly utilised as a cost- and time-efficient method of SNP discovery and genotyping in salmonid genomic studies, this map will provide a framework for orientation of the marker genotypes with the Atlantic salmon reference genome and putative candidate genes.

The large number of markers that can be discovered and scored in a single experiment is an advantage of the RAD-Seq approach. However, stringent filtering must be applied to avoid false positive SNPs, particularly in the recently duplicated salmonid genomes. We initially discovered > 28,000 putative SNPs in our dataset, but after removing putative PSVs and SNPs with excess missing genotypes or Mendelian errors, only *ca.* 8,000 SNPs remained for linkage map construction. The large number of missing genotypes in the dataset is partially due to a degree of irregularity in the sequence coverage across individuals and mapped RAD sites. SNPs removed at this stage were mainly from the PE contigs where sequence coverage is inevitably lower than at RAD loci. Post-filtering, the average genotyping rate in the dataset across 77 individuals and ~8,000 SNPs was 76%. This is a substantial increase in the average genotyping rate in the unfiltered data (*ca.* 50%; Figure 1). However, given the relatively small sample size in this study, any missing data will reduce the resolution of the maps constructed and reported. There is a strong relationship between sequence coverage and proportion of successful SNP genotype calls across individuals (Figure 1). This is despite ensuring near equal quantities of offspring genomic DNA in each library. Therefore, to avoid high proportions of missing genotypes in future experiments using RAD-Seq, it is important to (i) strive for identical quantity and quality of input genomic DNA per individual and (ii) to account for the uneven read distribution across individuals and scale up the projected read coverage per individual accordingly.

**Figure 5:**
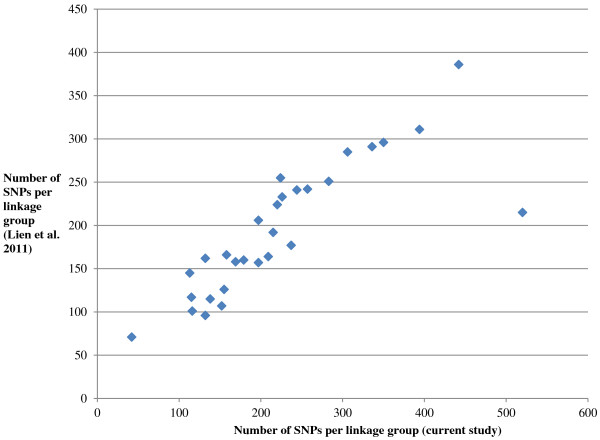
**Comparison of the number of SNPs per linkage group with a previously published map.** The number of SNPs per linkage group (assigned using the CRI-MAP software) in the current study was plotted against the number of SNPs per linkage group in the SNP map study conducted by Lien et al. [11]. The number of SNPs identified per linkage group in the two studies were highly correlated (r = 0.83).

The number of SNPs initially assigned to each linkage group using the CRI-MAP software and anchor marker information in this study was compared to the SNP linkage map constructed by Lien et al [11] and a strong positive correlation was observed (Figure 5). This is despite the use of different sequencing and genotyping technologies for the construction of the maps. This suggests that SbfI RAD-Seq is yielding an unbiased sample of the salmon genome, and that the number of SNPs per linkage group in both studies is likely to be related to chromosome size. Dense sex-specific microsatellite linkage maps (ASalBase, [41]) are available for each of the four parents in the two SalMap families [8]. In the current study, previously mapped markers were used as anchors in order assign RAD-Seq SNPs to salmon linkage groups, thus allowing a partial integration of the existing linkage maps with a dense SNP linkage map. To our knowledge, our study is the first to attempt this in Atlantic salmon. Due to constraints in the sample size and the different properties associated with the inheritance of the different marker types, we were unable to produce a reliable combined anchor marker-SNP linkage map using the Onemap software. Therefore, final maps are comprised only of RAD-Seq derived SNP markers. For tightly linked SNPs, the number of recombination events is likely to be small in families of this size (i.e. *ca.* 40 individuals). While we did not observe more than 25% of markers segregating as a single unit on linkage maps (except for the case of the putative centromeric regions in male maps), an improved reliability of marker order may be obtained by analysing additional families with larger numbers of offspring to increase the number of informative meioses.

The length of genomic DNA sequenced at each RAD locus, including the RAD locus itself and the PE contig, is approximately 500 bp. Therefore, multiple SNPs are frequently observed at a single locus and recombination between these SNPs is unlikely. As such, these SNPs are expected to map to the same position on the linkage map. To test this, we analysed the positions of SNPs from RAD loci and PE contigs which originate from the same restriction cut site within the genome. We identified 26 restriction cut sites with mapped SNPs from both the RAD locus and PE contig. In ~60% of cases, SNPs from the RAD locus and associated PE contig mapped to the same cM position on the map. Where this did not occur, PE SNPs were found to be positioned at the terminal ends of linkage groups. Given the lower read coverage for the PE contig due to the nature of the RAD-Seq protocol, SNPs derived from the PE contig may have a higher genotyping error rate than those from the RAD locus. A common feature of linkage mapping software is the positioning of markers with higher error rates at the ends of linkage groups, which could explain the instances where RAD loci and PE contig derived SNPs did not co-localise on linkage maps.

### Map lengths and recombination ratios

The large difference in recombination rate between Atlantic salmon males and females, and the distribution of the recombination events along the chromosome, have been a subject of much discussion in the literature [9-11,22]. We observed only a relatively small difference in overall map length in the current study (~1:1.5) which is comparable to that observed in the SNP linkage map of Lien et al. [11] (1:1.38). Also in common with previous studies, we identified an increased clustering of male-segregating markers compared to females (e.g. Figure 3). Therefore, this study supports the hypothesis that the major difference between male and female Atlantic salmon is in the position of recombination events on the chromosome and not the overall frequency of recombination events. However, it should be noted that since sex specific markers were used, there were no common SNPs between the male and female maps within a family. Therefore, our interpretations of the map distance differences and recombination events are based on overall patterns of linkage group size and marker clustering, rather than direct comparisons between marker pairs.

### Comparative genomics

Approximately 17% of the SNPs had flanking sequence data which gave a significant alignment with an annotated gene in the stickleback genome. Including the additional step of aligning the mapped SNP flanking sequences to the salmon reference genome contigs increased the proportion of mapped SNPs associated with a putative gene to *ca.* 50%. There is a GC bias in the SbfI recognition sequence (5′ CCTGCA/GG 3′ and 3′ GG/ACGTCC 5′) which may result in a higher-than-expected frequency of SbfI cut sites within gene-rich regions of the genome. This bias in SbfI cut sites in potentially gene-rich regions of the genome has been observed in other SbfI RAD-Seq studies [46]. In total, Atlantic salmon orthologs for 2,030 stickleback genes were identified. On average, over half the genes identified on a linkage group were unique to a single individual map, with approximately a third of genes being mapped in two mapping parents. A substantial overlap between individuals in genes identified may not be expected since maps were not built using common SNP markers.

Gene-associated mapped SNPs were then used to investigate the orthology between the Atlantic salmon and the stickleback genome. Due to the large evolutionary distance between stickleback and salmon, extensive chromosomal rearrangements are likely to have occurred in both species. The stickleback genome contains 21 chromosomes (annotated as linkage groups in Ensembl), which is fewer than Atlantic salmon (*viz.* 29, equal to the number of linkage groups). Most salmon linkage groups were assigned to at least one stickleback group (see Materials and methods), with three salmon linkage groups (5, 19 and 22) remaining unassigned, possibly due to the lower number of gene-associated markers on these linkage groups. Twelve stickleback linkage groups aligned to more than one Atlantic salmon linkage group, with one (stickleback linkage group 20) aligning to three Atlantic salmon linkage groups (Table 4, column 5; summarised in Additional file 1: Table SA4). These orthologies are consistent with published literature [11], and may represent homeologous chromosomes within the Atlantic salmon genome.

Using microsatellites from the same mapping families as the current study, Danzmann et al. [6] were able to identify homeologous regions within the Atlantic salmon genome (defined by shared duplicated markers). As well as this, they characterised the ancestral proto-Acinopterygian karyotype which is hypothesised to have had 13 linkage groups (labelled A - M), and assigned each Atlantic salmon linkage group as originating from one or more of these ancestral linkage groups. The Atlantic salmon homeologies given in Table 5 were based on shared Atlantic salmon genome contigs which were assigned to linkage groups by Blastn alignment to mapped RAD contigs. The Atlantic salmon genome assembly used is the first published draft, and may contain assembly errors. Therefore it is possible that the presence of chimeric or repetitive contigs could create spurious homeologies between linkage groups. 70% of the homeologies given in Table 5 also contained regions of common ancestral origin based on the ancestral karyotype (linkage groups labelled A - M) as characterised by Danzmann et al. [6]. We further confirmed these Atlantic salmon homeologies by looking for the Atlantic salmon linkage groups which have a stickleback linkage group in common (Table 4; summarised in Additional file 1: Table SA4). Of the 12 homeologies described in Additional file 1: Table SA4, 8 have been described in a study conducted by Danzmann et al. [8], and 7 have been described in Phillips et al. [44]; these have been highlighted in Table 5.

Linkage group orthologies between Atlantic salmon and rainbow trout were also analysed using published studies [6,44] to confirm the ancestral genome duplication within the salmonids relative to other teleost fish genomes. Overall, a 1:1 correspondence between Atlantic salmon and rainbow trout linkage groups was not seen (Table 4). This may be explained by the genomic rearrangements that have occurred in the two different genomes post-diploidisation. However, two Atlantic salmon linkage groups sharing a single stickleback linkage group (Atlantic salmon homeologous linkage groups) were seen to map to two rainbow trout linkage groups (apart from two cases where three rainbow trout linkage groups were found for two Atlantic salmon linkage groups). This 1:2:2 correspondence between stickleback, Atlantic salmon and rainbow trout linkage groups respectively supports the salmonid specific ancestral genome duplication.

## Conclusions

In this study we used paired-end RAD-Seq to generate a high density SNP linkage map of the Atlantic salmon genome in an outbred population. The pattern of recombination between male and female mapping parents revealed a difference in the distribution of putative recombination events across the linkage groups. Comparative genomics allowed the identification of genes proximal to (or containing) the mapped RAD-Seq SNPs. Homeologous regions within the Atlantic salmon genome and the putative orthologues of the salmon linkage groups in the stickleback and rainbow trout genome were identified and confirmed, providing support for a salmonid specific genome duplication. RAD-Seq is an increasingly popular tool for QTL mapping and population genomics, and this new map will provide a useful framework for future genomics studies.

## Materials and methods

### RAD library preparation and sequencing

The two SalMap families (Br5 and Br6) [8] used in this study are from a salmonid genetics resource population, and studies using these samples have been previously published [5,6,11,44,47-49]. Therefore, no new fish experiments or sampling was carried out for this study. DNA samples for these fish were obtained, and for each family, both parents and 46 offspring were sequenced using RAD-seq (total n = 96). Genomic DNA samples from each individual were quantified using spectrophotometry (Nanodrop) and checked for genomic integrity by agarose gel electrophoresis. A total of eight RAD libraries were prepared, with two parent libraries (n = 2) and six offspring libraries (each n = 14 - 16; Additional file 1: Table SA1). Since each library was subsequently sequenced in an individual lane, this design ensured higher sequence coverage of the parents compared to the offspring (Additional file 1: Table SA1). RAD libraries were prepared according to the methodology described in Etter et al. [50] with modifications as described in Houston et al. [38]. Briefly, each sample (1.5 μg DNA per sample for parent libraries/0.25 μg DNA per sample for offspring libraries) was digested with SbfI-HF (NEB) (recognition cut site 5′ CCTGCA/GG 3′ and 3′ GG/ACGTCC 5′). Within each library, a P1 adaptor containing an individual-specific nucleotide barcode was ligated to the gDNA of each sample. Details of the library composition and nucleotide barcodes are given in Additional file 1: Table SA1. The samples within each library were then pooled and the pools were sheared to ~400 bp fragments using a Covaris S2 sonicator (Covaris Inc., Woburn, USA), then size selected through agarose gel electrophoresis to an approximate range of 250 - 500 bp. The P2 adapter, a “Y” adapter with divergent ends, was ligated to the fragments and the libraries underwent 18 cycles of PCR amplification followed by a final size selection for the 300 - 500 bp fraction of the fragments. Size ranges of the completed libraries were verified using electrophoresis (Agilent Bioanalyser) and concentrations were determined using spectrophotometry (Nanodrop). Each library was sequenced on an individual lane of the Illumina HiSeq 2000 at the Edinburgh Genomics Facility, University of Edinburgh (https://genomics.ed.ac.uk/). Raw sequences are available from the ENA (http://www.ebi.ac.uk/ena/), accession number PRJEB4502.

### RAD-Seq bioinformatic pipeline and SNP calling

The process for generating SNP genotype data for the individuals in the RAD-Seq experiment was as follows. Firstly, raw reads were ‘demultiplexed’ and assigned to individual samples according to their nucleotide barcode using RADpools v1.2.1 [51], resulting in an individual fastq file per animal. The raw reads from each of these individuals were then merged into populations and the consensus sequence at each side of the SbfI cleavage sites were generated using ustacks and cstacks v0.992 [52], allowing a mismatch of 1 base only. The assembly of the paired-end sequences at each SbfI flanking site was performed with clc assembly cell v3.22. The paired-end reads were aligned back to the assembly using stampy 1.0.13 [53]. PCR duplicates were detected with Picard MarkDuplicates v1.55 (http://picard.sourceforge.net/) and excluded. Overall, 482,547 consensus RAD contigs were generated, of which 366,219 were from the RAD loci and 116,328 were from the PE contig. 2% of RAD loci had more than one PE contig associated with it. SNPs were called using samtools v0.1.18 [54] and then filtered using vcfutils to ensure a minimum overall read count at the locus of 500, a maximum of 2,000 (to help exclude potential repeat regions) and a SNP quality score of 60. Only genotypes with a quality score of 20 and a depth of 6 reads were used; the others were assigned no call. Only SNPs with a call in both parents of a family were retained for further analyses.

### SNP genotype quality control and filtering

The RAD-Seq bioinformatics pipeline described above resulted in a set of 28,415 putative SNPs in both single and paired-end consensus sequences. Due to variation in sequence coverage between individuals there was a large number of missing genotypes in the dataset (see Figure 1). Paralogous sequence variants (PSVs) within duplicate regions of the genome with a very high sequence similarity will be retained by the pipeline above. Therefore, filtering to remove (i) individuals and SNPs with excess missing data (RQTL software, http://www.rqtl.org/), (ii) putative PSVs (inferred by excess heterozygosity) (Plink software, http://pngu.mgh.harvard.edu/~purcell/plink/) and (iii) apparent Mendelian errors (VIPER software, http://bioinformatics.roslin.ac.uk/viper/) was carried out. Given parental genotypes, a Mendelian error was defined as a highly improbable offspring genotype at a given SNP. Removing SNPs with genotypes in fewer than half of the individuals left 13,637 SNPs. Removing individuals with poor genotyping coverage (genotyped at fewer than 25% of the SNPs) resulted in the removal of 15 individuals (Figure 1). SNPs showing excess (> 70%) heterozygosity across the two families used in this mapping study (n = 96) were removed from the dataset as putative PSVs, leaving 8,742 SNPs. Four individuals with > 200 genotypes defined as Mendelian errors were removed from the analysis. 485 SNPs with two or more Mendelian errors were discarded. SNPs with one Mendelian error (603 SNPs) were set to missing for the genotype in question. The final dataset remaining for linkage map construction consisted of 77 individuals (36 offspring and 2 parents in Br5; 37 offspring and 2 parents in Br6) and 8,257 SNP markers, with an overall genotyping rate of 76.2%. The SNP filtering procedure is summarised in Table 1.

### Linkage map construction

Linkage maps were constructed separately for the four parents in the two mapping families using a two-step process. In step 1, the SNP markers were clustered into putative linkage groups using 116 markers from a pre-existing study genotyped across the SalMap families [8] as anchors (including microsatellites, minisatellites, allozymes and SNPs; Additional file 1: Table SA2). Markers were chosen so that at least one informative marker was present in each linkage group. The two-point linkage between all markers was then calculated using the ‘twopoint’ option of the CRI-MAP software (version 2.4) as modified by Xuelu Liu (Monsanto) [42]. SNP markers were then assigned to linkage groups using the ‘autogroup’ option and mapped anchor markers (Additional file 1: Table SA2), starting at a LOD of 40 and applying a stepwise decrease in LOD score threshold to a minimum of 4. For each SNP, the segregation type (aaXaa; aaXbb; aaXab; abXaa; abXab) of the SNP in each family was determined and SNPs showing informative segregation patterns for linkage map construction (aaXab – female segregating marker; abXaa – male segregating marker) were identified. The best estimated order of SNPs on each linkage group was calculated using the ‘order.seq’ algorithm of the Onemap software [43], modified for parallelised computing by Marcelo Mollinari (Department of Genetics, University of São Paulo) with the following parameters: n.init = 5, THRES = 4, draw.try = FALSE. SNP marker order was confirmed as the best order using the ‘ripple.seq’ function of Onemap with a word size of 7 and applying a LOD threshold of 4.

The map position in centiMorgans (cM) was calculated according to the Haldane mapping function. Marker genotypes containing errors can appear as recombination events and result in erroneous positioning of SNPs some distance removed from other markers at the ends of linkage groups. Therefore, maps for each parent and each linkage group were investigated manually, and SNPs with a gap of greater than 30 cM in male maps and 20 cM in female maps from the neighbouring SNP at the ends of the linkage groups were removed. Maps were drawn using the MapChart software [55] (an example linkage group is shown in Figure 2).

### Recombination ratio calculation and comparing marker distributions

Recombination ratios between males and females could not be directly estimated using marker intervals since male and female maps within families did not contain the same markers. Instead, recombination ratios were estimated by comparing map lengths. In order to compare the distribution of markers between the sexes, linkage groups were split into intervals of equal size and the distribution of the markers across the intervals was compared for males and females across linkage groups. To define the intervals, the shorter of the parental maps was split into 5 cM intervals. If map length was not long enough to produce more than five distinct intervals then a smaller cM interval was chosen in order to split the map into 5 equally sized intervals. The longer parental map was then split into an equal number of intervals. For example for family Br5 and linkage group 1, the male map was shorter than the female map (85 cM and 229 cM respectively). Therefore, the male map was split into 18 intervals of 5 cM. The female map was then split into 18 marker intervals of equal size (18 x ~13 cM) and the percentage of SNPs mapping to each interval for both sexes was calculated. For each linkage group and each sex across both families, the five intervals with the highest percentage of markers were identified, and the averages of these percentages are given in Figure 3.

### Alignment of RAD loci with Atlantic salmon reference genome

In order to assign Atlantic salmon reference genome contigs to linkage groups, the mapped RAD contigs were first repeat-masked using the RepeatMasker software using the “Salmon Raw Repeat DB v1.6” database (http://web.uvic.ca/grasp/). Masked sequences were then aligned with the Atlantic salmon reference genome (NCBI Assembly GCA_000233375.1; http://www.ncbi.nlm.nih.gov/Traces/wgs/?val=AGKD01) using Blastn with an E-value threshold of 1e^-30^ for RAD loci and 1e^-80^ for PE contigs. RAD loci or PE contigs aligning to multiple (> 2) reference genome contigs were excluded.

### Comparative genomics: identification of SNP-associated putative genes

All known three-spined stickleback gene nucleotide sequences were downloaded from Ensembl BioMart (http://www.ensembl.org/biomart/martview/, Database = Ensembl Genes 72, Dataset = *Gasterosterus aculeatus* genes (BROADS1)). The identification of SNPs within or close to putative genes was performed in two stages. In the first stage, the mapped RAD contigs were screened for sequence similarity to stickleback (*Gasterosterus aculeatus*) genes using tblastx (E-value < 1e^-5^). tblastx was chosen as it is more sensitive to protein homologies between distantly related species using sequence data (for example [56,57]), since it translates sequences in all three frames before alignment, thus overcoming problems of detecting open reading frames in the context of frame shifts between species. In the second stage, the linkage group assigned, repeat-masked *Salmo salar* reference genome contigs were aligned against the stickleback gene sequences. This was done in order to detect mapped RAD-Seq SNPs which are in close proximity to a stickleback gene ortholog (tblastx, E-value < 1e^-5^). Only the two most significant alignments were retained in an attempt to avoid spurious alignment to multiple genes from different stickleback linkage groups (for example due to similarity of genes from the same gene family).

### Comparative genomics: synteny analysis and assignment of Atlantic salmon linkage groups to stickleback linkage groups

As described above, for each linkage group and each individual separately, RAD loci and PE contigs associated with mapped SNPs were aligned against the stickleback gene sequences. RAD loci and PE contigs were then grouped into a single RAD locus in order to be counted only once (so as to prevent bias in the stickleback linkage group identified as syntenic to the Atlantic salmon linkage group) and the total number of RAD loci showing significant alignment to a gene on a particular stickleback linkage group was counted. For each Atlantic salmon linkage group, a single synteny relationship was assigned only if the number of significant alignments for a particular stickleback linkage group was twice (or more) than the number of significant alignments for any other stickleback linkage group, and this relationship was seen in all mapping parents. The only exceptions to this was in cases where two stickleback linkage groups showed identical numbers of RAD loci alignments, then both were assigned to that Atlantic salmon linkage group.

### KASP assay genotyping

To verify a subset of the RAD-Seq SNPs and to investigate the correspondence between individual genotypes obtained by RAD-Seq and those obtained by the more established ‘KASP’ technology (LGC Genomics, Herts, UK; http://www.lgcgenomics.com/genotyping/kasp-genotyping-reagents/), a subset of RAD-Seq SNPs with flanking sequence (Additional file 3) were submitted to LGC Genomics for KASP assay design and genotyping of the two SalMap families (n = 96) [8]. A total of 55 KASP assays were designed, of which eight returned monomorphic data across the 96 samples. Therefore, genotypes for 47 polymorphic SNPs (38 from the RAD loci, 9 from the PE consensus) from 41 RAD sites (Additional file 3) were investigated for correspondence of scored genotypes between the two genotyping techniques in the same samples.

### Availability of supporting data

The raw RAD sequence reads supporting this article are available in the European Nucleotide Archive (ENA) repository, [PRJEB4502, http://www.ebi.ac.uk/ena/].

## Competing interests

The authors declare that they have no competing interests.

## Authors’ contributions

Conceived and designed the experiments: RDH, SCB. Prepared RAD libraries: NL. Managed sequencing of RAD libraries: KG. Analysed data: SG, TC. Wrote the paper: SG, RDH. All authors read and approved the final manuscript.

## Supplementary Material

Additional file 1 **This file contains Supplementary Tables A1-A4. Table A1** – Library structure and read depth for the paired-end RAD-sequencing libraries. **Table A2** – Markers used as anchors in CRI-MAP for assignment of RAD-derived SNPs to linkage groups, and their corresponding Atlantic salmon linkage groups/chromosomes. **Table A3** – Map length (cM) for each mapping parent and the comparison between the sexes. **Table A4** – Homeologous Atlantic salmon linkage groups with the stickleback and rainbow trout linkage groups and proto-Acinopterygian linkage groups which they have in common. Click here for file

Additional file 2 **Putative PSVs: Variants removed from analysis due to excess heterozygosity.** Contains the following information for each PSV identified: ID of the SNP identified as a PSV (column 1); RAD consensus ID (column 2) and RAD consensus sequence (column 6) from which the SNP originates; position of the SNP identified as a PSV in the RAD consensus sequence (bps) (column 3); SNP alleles (columns 4 and 5). Click here for file

Additional file 3 **SNPs used for RAD-Seq validation. Contains ID and sequence information for the 47 SNPs validated by KASP technology.** For each SNP, the percentage concordance between the genotypes obtained from RAD-Seq and those obtained from the KASP technology for the two SalMap families is given in column 2. Click here for file

Additional file 4 **Linkage maps.** Annotated with alignment information to stickleback genes and Atlantic salmon genome contigs. Map distances are given in centiMorgans (cM). Each parent’s linkage map is given in separate sheets of the file. Click here for file

Additional file 5 4,367 Atlantic salmon reference genome contigs and the Atlantic salmon linkage groups to which they map. Click here for file

Additional file 6 112 reference genome contigs which mapped to more than one linkage group and the Atlantic salmon linkage groups to which they align. Click here for file
