# Supplementary material for: Linkage maps of the Atlantic salmon (Salmo salar) genome derived from RAD sequencing
Source: BMC Genomics. 2014 Feb 27;15:166. doi: 10.1186/1471-2164-15-166 (PMC4028894; doi:10.1186/1471-2164-15-166)
Supplement: Additional file 1 — This file contains Supplementary Tables A1-A4. Table A1 – Library structure and read depth for the paired-end RAD-sequencing libraries. Table A2 – Markers used as anchors in CRI-MAP for assignment of RAD-derived SNPs to linkage groups, and their corresponding Atlantic salmon linkage groups/chromosomes. Table A3 – Map length (cM) for each mapping parent and the comparison between the sexes. Table A4 – Homeologous Atlantic salmon linkage groups with the stickleback and rainbow trout linkage groups and proto-Acinopterygian linkage groups which they have in common. [file 1471-2164-15-166-S1.DOCX]

### Additional file 1 – Supplementary tables

This file contains Supplementary tables A1-A4

**Table A1 – Library structure and read depth for the paired-end RAD-sequencing libraries**

| Family Br5 | | | | | | Family Br6 | | | | | |
| --- | --- | --- | --- | --- | --- | --- | --- | --- | --- | --- | --- |
| Fish ID | Sex | Library | Barcode | No. PE Reads Raw * | No. Mapped Reads after PCR Duplicate Removal ** | Fish ID | Sex | Library | Barcode | No. PE Reads Raw * | No. Mapped Reads after PCR Duplicate Removal ** |
| Sire | M | 1 | CTAGG | 9,840,258 | 3,339,336 | Sire | M | 2 | CTAGG | 9,779,574 | 2,817,941 |
| Dam | F | 1 | GAGAT | 10,618,889 | 3,607,996 | Dam | F | 2 | GAGAT | 14,210,782 | 4,263,519 |
| Br5- 01 | F | 6 | CTAGG | 906,003 | 483,118 | Br6- 01 | F | 3 | CTAGG | 1,465,063 | 482,837 |
| Br5- 02 | F | 6 | GAGAT | 1,415,716 | 729,246 | Br6- 02 | M | 3 | GAGAT | 1,597,905 | 526,808 |
| Br5- 03 | F | 6 | GCGCC | 392,470 | 221,823 | Br6- 03 | M | 3 | GCGCC | 484,244 | 176,234 |
| Br5- 04 | M | 6 | GTACA | 2,227,594 | 1,132,308 | Br6- 04 | F | 3 | GTACA | 1,536,766 | 511,956 |
| Br5- 05 | M | 6 | GTGTG | 1,033,311 | 542,110 | Br6- 05 | M | 3 | GTGTG | 978,969 | 333,111 |
| Br5-06 | F | 8 | CATGA | 2,645,093 | 1,314,085 | Br6- 06 | F | 3 | TAGCA | 2,434,848 | 785,411 |
| Br5- 07 | M | 8 | CACAG | 2,994,367 | 1,462,823 | Br6- 07 | F | 3 | TCAGA | 1,787,224 | 593,222 |
| Br5- 08 | F | 6 | TCGAG | 1,265,152 | 663,088 | Br6- 08 | M | 3 | TCGAG | 1,549,776 | 525,250 |
| Br5- 09 | F | 6 | TGACC | 2,210,309 | 1,141,938 | Br6- 09 | M | 3 | TGACC | 2,051,583 | 687,784 |
| Br5- 10 | M | 6 | ACTGC | 2,071,445 | 1,078,076 | Br6- 10 | F | 3 | ACTGC | 2,359,457 | 785,385 |
| Br5- 11 | F | 6 | ACACG | 2,442,613 | 1,265,728 | Br6- 11 | M | 3 | ACACG | 2,049,384 | 706,676 |
| Br5- 12 | M | 6 | AGAGT | 2,429,375 | 1,244,671 | Br6- 12 | M | 3 | AGAGT | 2,694,450 | 855,237 |
| Br5- 13 | F | 6 | ATGCT | 3,168,094 | 1,591,906 | Br6- 13 | F | 3 | ATGCT | 2,617,124 | 854,990 |
| Br5- 14 | F | 7 | CTAGG | 1,235,965 | 268,147 | Br6- 14 | F | 3 | CAGTC | 2,166,291 | 735,312 |
| Br5- 15 | F | 7 | GAGAT | 1,469,717 | 313,862 | Br6- 15 | F | 3 | CATGA | 341,025 | 127,581 |
| Br5- 16 | M | 6 | CAGTC | 2,989,144 | 1,534,393 | Br6- 16 | M | 3 | CACAG | 601,453 | 212,743 |
| Br5- 17 | M | 6 | CATGA | 475,276 | 264,810 | Br6- 17 | F | 4 | CTAGG | 1,572,331 | 484,234 |
| Br5- 18 | M | 6 | CACAG | 542,851 | 299,218 | Br6- 18 | F | 4 | GAGAT | 1,984,142 | 597,852 |
| Br5- 19 | M | 7 | GCGCC | 690,542 | 164,317 | Br6- 19 | F | 4 | GCGCC | 430,374 | 148,146 |
| Br5- 20 | F | 7 | GTACA | 2,505,144 | 526,591 | Br6- 20 | M | 4 | GTACA | 1,925,850 | 589,276 |
| Br5- 21 | F | 7 | GTGTG | 1,096,955 | 236,014 | Br6- 21 | F | 4 | GTGTG | 1,194,498 | 368,664 |
| Br5- 22 | F | 7 | TAGCA | 1,941,267 | 421,905 | Br6- 22 | M | 4 | TAGCA | 2,804,243 | 813,251 |
| Br5- 23 | F | 7 | TCAGA | 1,831,912 | 393,314 | Br6- 23 | M | 5 | CTAGG | 1,278,277 | 754,333 |
| Br5- 24 | M | 7 | TCGAG | 1,564,190 | 341,612 | Br6- 24 | F | 5 | GAGAT | 1,450,505 | 847,563 |
| Br5- 25 | F | 7 | TGACC | 2,716,325 | 589,106 | Br6- 25 | M | 4 | TCAGA | 2,094,522 | 634,384 |
| Br5- 26 | M | 7 | ACTGC | 1,910,822 | 417,196 | Br6- 26 | M | 4 | TCGAG | 1,683,378 | 522,012 |
| Br5- 27 | F | 7 | ACACG | 2,599,942 | 577,493 | Br6- 27 | F | 4 | TGACC | 2,914,441 | 897,761 |
| Br5- 28 | F | 8 | CTAGG | 1,734,761 | 828,686 | Br6- 28 | F | 4 | ACTGC | 1,919,675 | 597,518 |
| Br5- 29 | M | 7 | AGAGT | 2,672,124 | 566,127 | Br6- 29 | M | 4 | ACACG | 2,211,708 | 698,042 |
| Br5- 30 | F | 8 | GAGAT | 2,109,042 | 972,312 | Br6- 30 | M | 4 | AGAGT | 3,916,489 | 1,110,022 |
| Br5- 31 | F | 8 | GCGCC | 687,604 | 361,519 | Br6- 31 | F | 4 | ATGCT | 2,187,938 | 669,231 |
| Br5- 32 | M | 7 | ATGCT | 3,038,521 | 636,525 | Br6- 32 | M | 4 | CAGTC | 2,214,529 | 689,992 |
| Br5- 33 | M | 7 | CAGTC | 2,292,598 | 501,144 | Br6- 33 | F | 5 | GCGCC | 714,044 | 443,931 |
| Br5- 34 | M | 7 | CATGA | 466,226 | 111,114 | Br6- 34 | F | 5 | GTACA | 2,251,290 | 1,299,516 |
| Br5- 35 | M | 7 | CACAG | 750,420 | 174,430 | Br6- 35 | M | 5 | GTGTG | 1,308,700 | 767,835 |
| Br5- 36 | M | 8 | GTACA | 2,491,872 | 1,157,827 | Br6- 36 | M | 5 | TAGCA | 2,405,386 | 1,401,132 |
| Br5- 37 | M | 8 | GTGTG | 1,507,512 | 718,618 | Br6- 37 | F | 5 | TCAGA | 2,752,922 | 1,570,525 |
| Br5- 38 | F | 8 | TAGCA | 2,531,598 | 1,209,215 | Br6- 38 | M | 5 | TCGAG | 1,749,406 | 1,031,150 |
| Br5- 39 | M | 8 | TCAGA | 3,370,670 | 1,556,289 | Br6- 39 | M | 5 | TGACC | 2,789,074 | 1,614,461 |
| Br5- 40 | F | 8 | TCGAG | 2,652,488 | 1,252,618 | Br6- 40 | F | 5 | ACTGC | 2,945,707 | 1,701,816 |
| Br5- 41 | M | 8 | TGACC | 3,505,350 | 1,664,949 | Br6- 41 | F | 5 | ACACG | 3,268,172 | 1,876,396 |
| Br5- 42 | M | 8 | ACTGC | 3,036,980 | 1,453,001 | Br6- 42 | M | 5 | AGAGT | 3,030,322 | 1,749,408 |
| Br5- 43 | F | 8 | ACACG | 4,186,888 | 1,987,317 | Br6- 43 | F | 5 | ATGCT | 4,074,713 | 2,283,001 |
| Br5- 44 | F | 8 | AGAGT | 3,677,255 | 1,727,430 | Br6- 44 | F | 5 | CAGTC | 3,336,800 | 1,931,108 |
| Br5- 45 | M | 8 | ATGCT | 4,126,124 | 1,919,761 | Br6- 45 | M | 5 | CATGA | 614,665 | 380,262 |
| Br5-46 | M | 8 | CAGTC | 3,457,228 | 1,654,626 | Br6- 46 | M | 5 | CACAG | 696,853 | 426,350 |

## * Number of Illumina paired-end reads per individual following demultiplexing of reads

** Number of reads following removal of ‘PCR duplicates’ (paired-end reads with identical read 1 and read 2) which should approximate the number of unique DNA fragments in the sample (see Davey et al. 2012).

**Table A2 – Markers used as anchors in CRI-MAP for assignment of RAD-derived SNPs to linkage groups, and their corresponding Atlantic salmon linkage groups/chromosomes**

| **Atlantic salmon Linkage group** | **Atlantic salmon Chromosome** | **Marker name** | **Marker type҂** | **Marker Information§** |
| --- | --- | --- | --- | --- |
| 1 | 2 | Omy11/1INRA  OmyFGT8/1TUF  Oneµ18  Ssa202  Ssa406UOS  Ssa-A14/1  Ssa-A15/1  Str4/1INRA | Microsatellite  Microsatellite  Microsatellite  Microsatellite  Microsatellite  Minisatellite  Minisatellite  Microsatellite | 1  2  U56718.1  U43695.1  AJ402723.1  Unpublished  Unpublished  3 |
| 2 | 10 | Ogo8  Oneµ5  Ssa-A13  Str-A3/2 | Microsatellite  Microsatellite  Minisatellite  Minisatellite | AF009780  U56704  Unpublished  4 |
| 3 | 14 | Ssa0014ECIG  Ssa0033ECIG  Ssa0169bECIG | SNP  SNP  SNP | 119096977  119096998  119097160 |
| 4 | 6 | Omy27/1INRA  OmyFGT1TUF  OmyRGT30/2TUF  Ssa171  Ssa-A12 | Microsatellite  Microsatellite  Microsatellite  Microsatellite  Minisatellite | 1  2  2  U43693.1  Unpublished |
| 5 | 13 | Sfo23  Ssa420UOS  SSsp2201  Str-A5  Str-A8/1 | Microsatellite  Microsatellite  Microsatellite  Minisatellite  Minisatellite | 5  AJ402737.1  AY081807.1  6  Unpublished |
| 6 | 12 | Omy11/2INRA  Omy21INRA  Omy27DU  OmyFGT25TUF  OmyRGT35TUF  SSsp2210 | Microsatellite  Microsatellite  Microsatellite  Microsatellite  Microsatellite  Microsatellite | 1  1  7  2  AB087604.1  AY081808.1 |
| 7 | 24 | Ocl9  SSsp2215  SSsp2216 | Microsatellite  Microsatellite  Microsatellite | AF028698  AY081810.1  AY081811.1 |
| 8 | 15 | Omy27/2INRA  Omy301UoG  Oneµ9  Ssa197  Ssa401UOS  Ssa-A60  Str-A3/1  Str-A8/2  Str-A8/3 | Microsatellite  Microsatellite  Microsatellite  Microsatellite  Microsatellite  Minisatellite  Minisatellite  Minisatellite  Minisatellite | 1  8  U56709.1  U43694.1  AJ402718.1  6  4  Unpublished  Unpublished |
| 9 | 11 | Ssa132  Ssa408UOS  Ssa413UOS  SSspG7 | Microsatellite  Microsatellite  Microsatellite  Microsatellite | U58901.1  AJ402725.1  AJ402730.1  AY081813.2 |
| 10 | 9 | MST541INRA  Ogo2/2  OmyFGT21TUF  OmyRGT30/1TUF  Oneµ7  Ssa412UOS  Ssa45/2micUOS  Ssa-A33  Ssa-A34/2  Str85INRA | Microsatellite  Microsatellite  Microsatellite  Microsatellite  Microsatellite  Microsatellite  Microsatellite  Minisatellite  Minisatellite  Microsatellite | AB001072  AF009794  2  2  U56707.1  AJ402729.1  SRX000001  6  6  AB001059 |
| 11 | 3 | Oki2  OmyRGT32TUF  Ssa417UOS | Microsatellite  Microsatellite  Microsatellite | AF055428  AB087602.1  AJ402734.1 |
| 12 | 5 | Ocl2  Omy272/2UoG  OmyFGT8/2TUF  Ssa-A14/2  Ssa-A15/2  Str15INRA  Str4/2INRA  Str-A9/1 | Microsatellite  Microsatellite  Microsatellite  Minisatellite  Minisatellite  Microsatellite  Microsatellite  Minisatellite | AF028699  8  2  Unpublished  Unpublished  AB001058  3  6 |
| 13 | 19 | MC4R  Ssa289  Ssa407UOS  Ssa422UOS | SNP  Microsatellite  Microsatellite  Microsatellite | Unpublished  9  AJ402724.1  AJ402739.1 |
| 14 | 21 | Str-A22/1  Str-A22/2/1 | Minisatellite  Minisatellite | 6  6 |
| 15 | 27 | Ssa0122aECIG | SNP | 119097105 |
| 16 | 18 | OmyRGT55TUF  Ssa416UOS  Str-A12/1  Str-A9/2 | Microsatellite  Microsatellite  Minisatellite  Minisatellite | AB031201.1  AJ402733.1  Unpublished  6 |
| 17 | 1 | Ogo3  OmyRGT34TUF  Ssa14  Ssa410UOS  Str-A12/2  Str-A22/2/2 | Microsatellite  Microsatellite  Microsatellite  Microsatellite  Minisatellite  Minisatelllite | AF009795  AB031199.1  10  AJ402727.1  Unpublished  6 |
| 18 | 23 | OmyFGT16TUF  Ssa85  SSsp1605 | Microsatellite  Microsatellite  Microsatellite | 2  U43692.1  AY081812.1 |
| 19 | 8 | Ssa0136ECIG  Ssa0158ECIG | SNP  SNP | 119097123  119097148 |
| 20 | 25 | MEP-2* | Allozyme | 11 |
| 21 | 26 | IDDH-2*  OmyRGT44TUF  Ssa-A45/2/1 | Allozyme  Microsatellite  Minisatellite | 11  AB087611.1  6 |
| 22 | 17 | Ssa12  Ssa402/2UOS  Ssa404UOS | Microsatellite  Microsatellite  Microsatellite | U58900  AJ402719  AJ402721.1 |
| 23 | 16 | Ssa402/1UOS  Ssa403UOS | Microsatellite  Microsatellite | AJ402719  AJ402720.1 |
| 24 | 7 | Omy14INRA  Ssa418/1UOS  Ssa-A34/1 | Microsatellite  Microsatellite  Minisatellite | 1  AJ402735  6 |
| 25 | 20 | Ocl1/1  Oki10  Omy23INRA  OmyFGT14TUF  OmyFGT34TUF  Ssa421UOS  Ssa-A10 | Microsatellite  Microsatellite  Microsatellite  Microsatelllite  Microsatellite  Microsatellite  Minisatellite | AF028694  AF055435  1  2  2  AJ402738.1  6 |
| 28 | 4 | Ssa405UOS | Microsatellite | AJ402722.1 |
| 30 | 29 | Ogo4  Ssa-A11 | Microsatellite  Minisatellite | AF009796  6 |
| 31 | 28 | AAT-4*  SSA224 | Allozyme  Microsatellite | 11  AF019168.1 |
| 32 | 22 | Ssa419UOS  Ssa-A45/1 | Microsatellite  Minisatellite | AJ402736.1  6 |

҂SNPs were sourced from Moen et al., 2008.

§GenBank accession number provided if available. Otherwise a numbered reference is provided where applicable. Numbers correspond to the following references: 1=Gharbi et al. 2006. Genetics 172: 2405-2419; 2=Sakamoto, 1996, PhD thesis; 3=Krieg and Guyomard, Unpublished; 4=Prodöhl et al. 1994. Heredity 73: 556-566; 5=Angers et al. 1995. Journal of Fish Biology 47A 177-185; 6=Taggart et al. 1995. Animal Genetics 26:13-20; 7= Perry et al. 2001. Cytotechnology 37: 143-151; 8= Jackson et al. 1998. Heredity 80: 143-151; 9= O'Reilly et al. 1996. Canadian Journal of Fisheries and Aquatic Science 53: 2291-2298; 10= McConnell et al. 1995. Canadian Journal of Fisheries and Aquatic Science 52:1863-1872; 11= Wilson et al. 1995. Heredity 75: 578-588.

## Table A3 – Map length (cM) for each mapping parent and the comparison between the sexes

| **Atlantic salmon Linkage group** | **Atlantic salmon Chromosome** | **Br5 Female**  **(cM)** | **Br5**  **Male**  **(cM)** | **Ratio**  **M:F** | **Br6 Female**  **(cM)** | **Br6**  **Male**  **(cM)** | **Ratio**  **M:F** |
| --- | --- | --- | --- | --- | --- | --- | --- |
|  |  |  |  |  |  |  |  |
| 1 | 2 | 229 | 85 | 1:2.7 | 85 | 109 | 1.3:1 |
| 2 | 10 | 196 | 44 | 1:4.5 | 143 | 52 | 1:2.8 |
| 3 | 14 | 64 | 65 | 1:1 | 93 | 39 | 1:2.4 |
| 4 | 6 | 132 | 115 | 1:1.1 | 60 | 113 | 1.9:1 |
| 5 | 13 | 107 | 114 | 1.1:1 | 130 | 82 | 1:1.6 |
| 6 | 12 | 126 | 91 | 1:1.4 | 71 | 43 | 1:1.7 |
| 7 | 24 | 38 | 100 | 2.6:1 | 73 | 30 | 1:2.4 |
| 8 | 15 | 85 | 79 | 1:1.1 | 79 | 64 | 1:1.2 |
| 9 | 11 | 128 | 85 | 1:1.5 | 140 | 14 | 1:10 |
| 10 | 9 | 241 | 136 | 1:1.8 | 145 | 40 | 1:3.6 |
| 11 | 3 | 131 | 130 | 1:1 | 136 | 73 | 1:1.9 |
| 12 | 5 | 86 | 121 | 1.4:1 | 71 | 69 | 1:1 |
| 13 | 19 | 66 | 32 | 1:2.1 | 81 | 42 | 1:1.9 |
| 14 | 21 | 67 | 61 | 1:1.1 | 61 | 17 | 1:3.6 |
| 15 | 27 | 71 | 52 | 1:1.4 | 33 | 26 | 1:1.3 |
| 16 | 18 | 55 | 90 | 1.6:1 | 135 | 68 | 1:2 |
| 17 | 1 | 104 | 187 | 1.8:1 | 112 | 80 | 1:1.4 |
| 18 | 23 | 120 | 63 | 1:1.9 | 47 | 37 | 1:1.3 |
| 19 | 8 | 3 | 16 | 5.3:1 | 0 | 3 | NA |
| 20 | 25 | 13 | 22 | 1.7:1 | 26 | 16 | 1:1.6 |
| 21 | 26 | 59 | 17 | 1:3.5 | 45 | 23 | 1:2 |
| 22 | 17 | 65 | 31 | 1:2.1 | 108 | 44 | 1:2.5 |
| 23 | 16 | 114 | 55 | 1:2.1 | 56 | 54 | 1:1 |
| 24 | 7 | 93 | 85 | 1:1.1 | 67 | 80 | 1.2:1 |
| 25 | 20 | 96 | 50 | 1:1.9 | 89 | 75 | 1:1.2 |
| 28 | 4 | 69 | 112 | 1.6:1 | 77 | 90 | 1.2:1 |
| 30 | 29 | 97 | 44 | 1:2.2 | 16 | 17 | 1.1:1 |
| 31 | 28 | 85 | 22 | 1:3.9 | 78 | 0 | NA |
| 32 | 22 | 67 | 67 | 1:1 | 100 | 28 | 1:3.6 |
| **TOTAL** |  | **2,807** | **2,171** | **1:1.3** | **2,357** | **1,428** | **1:1.7** |

**Table A4 – Homeologous Atlantic salmon linkage groups with the stickleback and rainbow trout linkage groups and proto-Acinopterygian linkage groups which they have in common (Danzmann et al., 2008).** Atlantic salmon homeologies were defined depending on the number of

| **Atlantic salmon**  **Linkage groups** | **Stickleback**  **Linkage group** | **Rainbow trout**  **Linkage groups** | **Proto-Acinopterygian**  **Linkage groups** |
| --- | --- | --- | --- |
| 4/11 | 11 | 2/9 | E |
| 1/12/15 | 20 | 27/31/16 | B |
| 9/21 | 2 | 10/18 | J |
| 3/11 | 3 | 23/13 | M |
| 3/15 | 10 | 3/16 | B |
| 2/23 | 19 | 6/27 | M,J/K |
| 7/25 | 13 | 10/11,19 | I |
| 6/32 | 17 | 29/12 | L |
| 16/17 | 6 | 6/30 | D |
| 17/31 | 5 | 30/17,22 | D/E |
| 14/20 | 16 | 5/31 | C |
| 13/30 | 21 | 19/7 | M |
